# The Association Between Self-Reported Acquisition of Perinatal Knowledge and Perinatal Depressive Symptoms: Evidence from the Korean Early Childhood Education & Care Panel Study

**DOI:** 10.3390/epidemiologia7050145

**Published:** 2026-10-15

**Authors:** Hye Jung Cho, Hyeri Jung, Geum-Joon Cho, Jaehee Lee, Jeong Rim Lee, Hyun-Soo Park, Hee-Sun Kim

**Affiliations:** 1 Department of Obstetrics and Gynecology, Dongguk University College of Medicine, Goyang 10326, Republic of Korea; heajungcho@hanmail.net; 2 Department of Obstetrics and Gynecology, Dongguk University Ilsan Hospital, Goyang 10326, Republic of Korea; jhlpcjtkfkd@gmail.com; 3 Department of Obstetrics and Gynecology, Korea University College of Medicine, Seoul 02841, Republic of Korea; md_cho@hanmail.net; 4 Life Course Care Research Team, Korea Institute of Child Care and Education, Seoul 04535, Republic of Korea; leejaehee@kicce.re.kr; 5 Department of Child Development and Family Studies, Yonsei University, Seoul 03722, Republic of Korea; jeongttu@hanmail.net; 6 Family Medicine Residency, Providence St. Joseph Eureka Hospital, Eureka, CA 95501, USA; hsparkmd@gmail.com; 7 Department of Obstetrics and Gynecology, National Health Insurance Service Ilsan Hospital, Goyang 10444, Republic of Korea

**Keywords:** perinatal knowledge, postpartum depression, future childbirth intentions

## Abstract

Background/Objectives: Postpartum depression is a major public health concern in South Korea, where fertility is among the world’s lowest. We examined whether self-reported acquisition of perinatal knowledge is associated with perinatal depressive symptoms and whether these symptoms are related to intention for a further birth. Methods: We analyzed 3278 women from the nationally representative Korean Early Childhood Education & Care Panel Study, assessed with the Korean Edinburgh Postnatal Depression Scale (K-EPDS; screen-positive ≥ 11) at mid-pregnancy, late pregnancy, one month postpartum and the first annual follow-up (median child age 6 months). Acquisition of perinatal knowledge in four domains was self-reported at that follow-up. Associations were examined with logistic and linear regression and generalized estimating equations. Results: Screen-positive prevalence rose from 10.68% to 28.08% across the four assessments (OR 1.46 per assessment, 95% CI 1.41–1.53). Absence of reported knowledge on maternal–neonatal interaction was associated with screen-positive status in unadjusted analysis (OR 1.47, 95% CI 1.06–2.04) but not after adjustment for antenatal symptoms, social support and sociodemographic covariates (adjusted OR 1.10, 95% CI 0.67–1.80; *n* = 2037). Social support was inversely associated with screen-positive status (adjusted OR 0.64 per point, 95% CI 0.54–0.76). Among 2236 women with complete data, intention for a further birth was reported by 38.69% of never-symptomatic women, 35.92% with antenatal symptoms only, 28.68% with postpartum-onset symptoms and 23.51% with persistent symptoms (χ^2^ = 36.40, *p* < 0.0001). Conclusions: Social support, not reported perinatal knowledge, was independently associated with postpartum depressive symptoms, and postpartum-onset and persistent symptoms were associated with lower intention for a further birth. These findings support screening that begins in pregnancy and continues beyond the early postpartum period, together with strengthening social support; they do not establish that perinatal education prevents depressive symptoms.

## 1. Introduction

South Korea is facing an unprecedented demographic crisis, with one of the world’s lowest fertility rates—recorded at 0.75 in 2024—and a persistent downward trend over the past two decades [1]. According to recent national statistics, the average maternal age at first childbirth continues to rise, and the proportion of women reporting psychological difficulties during and after pregnancy has also increased significantly [1,2]. Against this backdrop, postpartum depression (PPD) has emerged as a major public health issue that adversely affects both maternal, neonatal, and childhood health. Previous studies have shown that PPD can lead to negative outcomes such as impaired mother–infant bonding, sociopsychological or neurodevelopmental delays in children [3,4]. Moreover, PPD has been linked to an increased risk of long-term physical and mental health disorders in mothers [5,6]. Considering the importance of maternal mental health, it is critical to identify modifiable factors that influence the onset and severity of PPD.

A growing body of research suggests that a lack of knowledge regarding childcare and prenatal management is associated with an increased risk of PPD [7]. Mothers who receive insufficient prenatal education may experience heightened stress, anxiety, and uncertainty, which can exacerbate depressive symptoms during the postpartum period. Furthermore, studies have indicated that prenatal education programs not only enhance maternal confidence but also play a crucial role in alleviating childbirth-related anxiety and reducing postpartum psychological distress [8,9]. Perinatal depressive symptoms also carry consequences beyond maternal wellbeing: in the Tohoku Medical Megabank Project, maternal psychological distress during pregnancy and depressive symptoms after delivery were each associated with a higher risk of autistic-related traits in toddlers among 23,218 mother–child pairs [10].

This study aims to comprehensively examine how self-reported acquisition of perinatal knowledge relates to postpartum depressive symptoms and subsequent childbirth intentions using data from the Korean Early Childhood Education & Care Panel Study (Korean ECEC Panel). By analyzing this nationally representative longitudinal dataset, we seek to assess how self-reported acquisition of perinatal knowledge is associated with maternal mental health, providing evidence on how Korean mothers acquire perinatal knowledge and how this relates to perinatal mental health. The primary hypothesis of this study was that the absence of reported knowledge on maternal–neonatal interaction is associated with screen-positive postpartum depressive symptoms (K-EPDS ≥ 11) at the first annual follow-up. Analyses of the remaining knowledge domains, factors associated with onset and remission, and of depressive-symptom trajectories in relation to childbirth intention were specified during revision and are reported as exploratory.

## 2. Materials and Methods

### 2.1. Study Design and Participants

**Figure 1:**
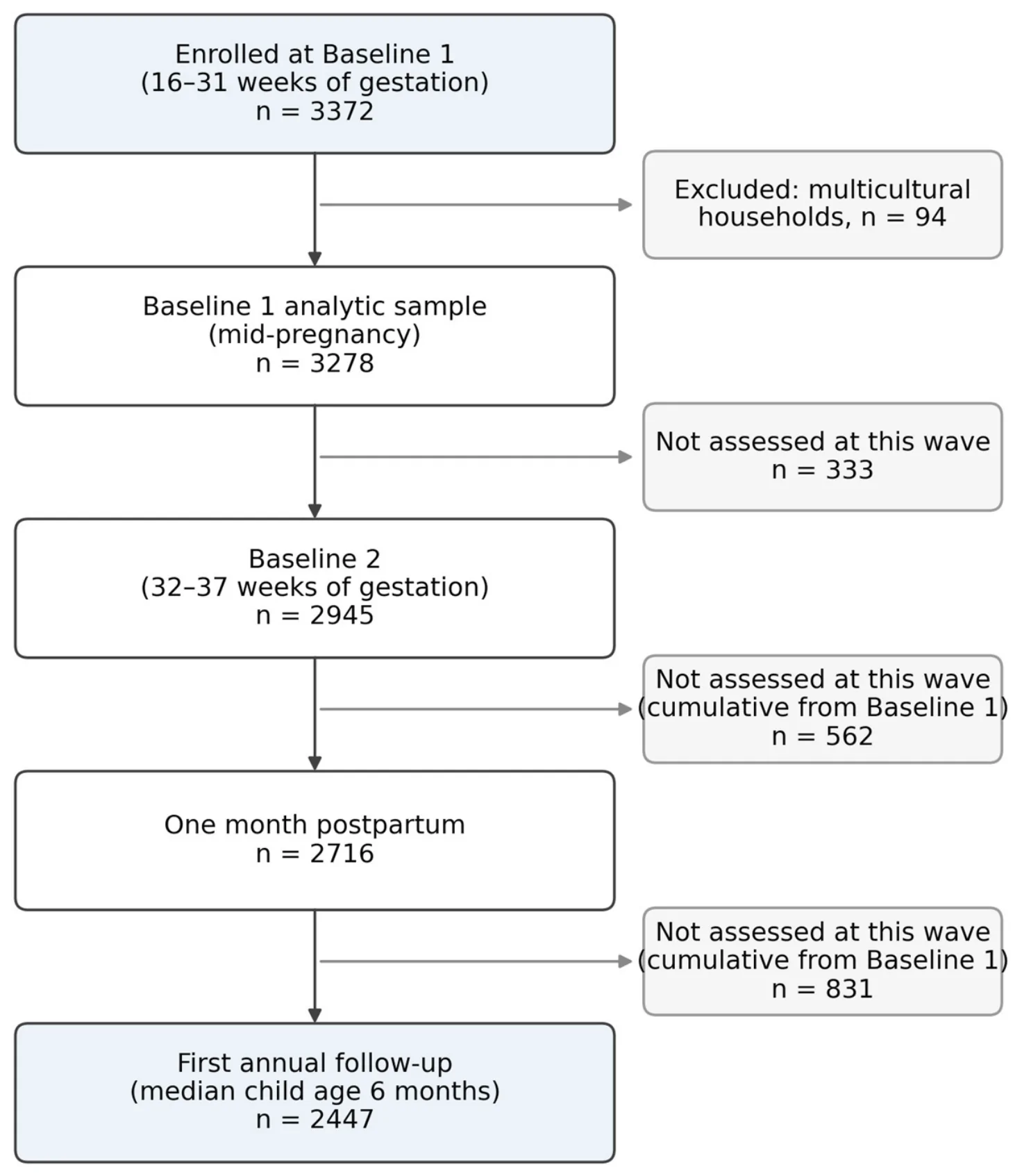
Participant flow through the four assessments of the Korean ECEC Panel Study.

This study utilized data from the Korean ECEC Panel. Pregnant women were enrolled to establish the panel, and data collection was conducted in multiple phases. The first baseline survey (Baseline 1) was conducted between September 2021 and February 2022 through a face-to-face TAPI (Tablet-Assisted Personal Interview) survey. The released dataset contains 3372 maternal records at Baseline 1 (gestational age 16–31 weeks); the panel’s published description of its recruitment reports 3380 women, and the eight-record difference reflects records not included in the released file. The second baseline survey (Baseline 2) was conducted between November 2021 and August 2022, with 3012 women (gestational age 32–37 weeks) through an online survey. Follow-up assessments included an online survey at one month postpartum and a home-visit TAPI survey at the first annual follow-up, conducted at a median child age of 6 months (range 3–12). Of the 3372 women enrolled at Baseline 1, 94 from multicultural households were excluded, leaving 3278 women in the analytic sample. The corresponding numbers were 2945 at Baseline 2, 2716 at one month postpartum, and 2447 at the first annual follow-up; 2236 women were assessed at all four time points. The derivation of the analytic sample is shown in Figure 1.

This study adhered to ethical standards and guidelines established for the Korean ECEC Panel.

### 2.2. Antepartum and Postpartum Depressive Symptoms Assessment

Depressive symptoms were assessed at all four time points using the Korean version of the Edinburgh Postnatal Depression Scale (K-EPDS), whose translation has been validated in Korean postpartum women (Cronbach’s α = 0.84) [11]. A score of 11 or higher was considered indicative of elevated depressive symptoms. This threshold was selected because an individual participant data meta-analysis of 58 studies (15,557 participants) reported that a cut-off of 11 or higher maximized combined sensitivity and specificity for detecting major depression among pregnant and postpartum women [12]. Because the EPDS is a screening tool rather than a diagnostic instrument, the outcome in this study was defined as high depressive symptoms rather than clinically confirmed depression. Scores obtained at Baseline 1 and Baseline 2 (during pregnancy) were classified as antenatal depressive symptoms, not PPD, as they were measured prior to delivery. Postpartum depressive symptoms were defined as a K-EPDS score of 11 or higher at one month postpartum or at the first annual follow-up. This study also analyzed risk factors associated with postpartum depressive symptoms and examined its relationship with subsequent pregnancy planning.

### 2.3. Measurement of Self-Reported Acquisition of Perinatal Knowledge

In Korea, education related to childbirth and infant care is delivered through several channels rather than a single standardized program. Antenatal classes are offered by obstetric hospitals and clinics, public health centers provide maternal and child health education under the national maternal and child health program, and postpartum care centers (sanhujoriwon), which are used by a majority of Korean mothers during the first weeks after delivery, routinely provide instruction on newborn care and maternal recovery. Participation in all of these is voluntary, and no national curriculum, standardized contact time or assessment of participant performance exists.

In the Korean ECEC Panel, this domain is captured by items asking, for each of four topics, where the mother obtained the relevant knowledge. The four topics are newborn care, maternal health management, promotion of interaction with the newborn, and prevention of sudden infant death. For each topic the respondent selects one of eleven options: hospital; midwifery clinic or postpartum care center; public health center or healthy family support center; childcare support center; publicly funded postpartum helper service; private postpartum helper; own parents; parents-in-law; siblings; learning through the internet or books; or never learned about the topic. A parallel item records whether the spouse attended.

These items therefore capture the source of knowledge acquisition rather than participation in a defined educational program, and they encompass both formal and informal sources. The exposure used in the present analysis was the response “never learned” for each topic, compared with all other responses. The items were administered in the first annual follow-up survey at a median child age of 6 months (range 3–12), and therefore concurrently with rather than before the postpartum outcome assessment. We therefore describe this variable throughout as self-reported acquisition of perinatal knowledge rather than as prenatal education. The distribution of reported sources is presented in Table S1. No information on curriculum content, contact hours, teaching method or participant assessment is available in the panel instrument. All four topics were analyzed, and the four corresponding indicator variables were entered simultaneously in the multivariable model. The parallel item on spousal attendance was not analyzed, because it is defined only among women who reported a formal source.

### 2.4. Data Processing and Variable Definition

The variables used to analyze the relationship between self-reported acquisition of perinatal knowledge and postpartum depressive symptoms were constructed from responses to the Korean ECEC Panel survey. As described in Section 2.3, the mother reported, for each of four content domains, where she had obtained her knowledge; the analyzed contrast was “never learned about this topic” versus any other response. The four domains are newborn care, which covers bathing, feeding, sleep and interaction with the newborn; maternal health management, which covers maternal health during pregnancy, physical and emotional changes after childbirth, and postpartum self-care; promotion of interaction with the newborn, which covers practices that foster bonding such as talking, singing and reading aloud; and prevention of sudden infant death. All four domains were entered simultaneously in the multivariable model.

To ensure consistency and minimize bias, responses with missing values were excluded using listwise deletion for multivariate models, and only participants with valid responses across baseline and follow-up surveys were retained for longitudinal analysis.

The variable was analyzed both cross-sectionally (at each time point) and longitudinally (tracking changes across pregnancy and up to the first annual follow-up).

Intention regarding a further birth was assessed at the first annual follow-up with a single item asking whether the mother planned to have another child. The panel instrument provides two response options: “yes (or currently pregnant)” and “no”. There is no “undecided” category. Of the 2447 women assessed, 839 (34.3%) reported intending a further birth and 1608 (65.7%) did not.

### 2.5. Statistical Analysis

Multivariate logistic regression models were constructed to identify factors independently associated with postpartum depressive symptoms and subsequent childbirth intentions.

Covariates included in the multivariable model were maternal age (under 30, 30–35, 36 or older), parity, plurality (singleton versus twin pregnancy), household income (low versus middle/high), residential region (metropolitan, mid-/small city, rural), pregnancy intention, a pre-pregnancy diagnosis of depression, maternal education, social support, and antenatal depressive symptom severity measured as the continuous K-EPDS score at Baseline 2. Social support was measured at the first annual follow-up with twelve items scored from 1 to 5 and was analyzed as the mean of the twelve items; in the analytic sample the mean was 4.03 (SD 0.68) with an observed range of 1.00 to 5.00 (*n* = 2629). Baseline 1 status could not be used as a covariate in the analysis of incident symptoms because the subgroup was defined by the absence of depressive symptoms at that assessment. NICU admission status was excluded as it may act as a mediator on the pathway from antenatal factors to postpartum depressive symptoms, and its inclusion would risk overadjustment.

Baseline associations were examined with Pearson’s chi-square test without continuity correction, and odds ratios are reported with Wald 95% confidence intervals. Change in screen-positive status across the four assessments was modeled with generalized estimating equations (binomial family, logit link, exchangeable working correlation, robust sandwich standard errors), fitted to all available assessments so that a woman contributes as many records as she completed. Assessment number was entered both as a linear term and, in a second model, as a categorical variable with Baseline 1 as the reference, to avoid imposing a linear trend in the log odds.

The primary exposure was absence of reported knowledge on maternal–neonatal interaction and the primary outcome was a K-EPDS score of 11 or higher at the first annual follow-up. All four knowledge domains were entered simultaneously. The same model was refitted with the continuous K-EPDS score as the outcome, in the identical sample and with the identical covariates, so that the two outcome specifications are directly comparable. A Benjamini–Hochberg false discovery rate correction was applied across the four knowledge domains; it was not applied to the other comparisons reported, which are exploratory.

Primary analyses were complete case. As a sensitivity analysis, participation in the first annual follow-up was modeled by logistic regression on Baseline 1 K-EPDS score, maternal age, low household income, parity, pregnancy intention and maternal education (*n* = 2945 with complete covariate data), and the main model was refitted with stabilised inverse-probability-of-participation weights. Weights among participants had a mean of 1.26 (SD 0.04), a range of 1.19 to 1.36 and a 99th percentile of 1.35; no truncation was applied. The analyses do not incorporate the panel’s own sampling or attrition weights.

The primary exposure and outcome, and the four depressive-symptom trajectory categories, were specified during revision of this manuscript and were not prespecified before the original analysis. All analyses other than the primary exposure–outcome association are therefore reported as exploratory.

Analyses were performed using SPSS version 27.0 (IBM Corp., Armonk, NY, USA) and were independently recomputed from the raw panel data files; the analysis script and its complete output are provided as non-published material. Statistical significance was set at *p* < 0.05.

During the preparation of this manuscript, the authors used Claude (Anthropic, San Francisco, CA, USA; 2026 releases) for language editing and for independent verification of the statistical estimates reported in Table 3, Table 4, Table 5 and Table 6 against the original panel data. The authors have reviewed and edited the output and take full responsibility for the content of this publication.

### 2.6. Ethics Statement

The Korean ECEC Panel was approved by the Institutional Review Board of the Korea Institute of Child Care and Education: the two antenatal baseline surveys under approval No. KICCEIRB-2021-09 (5 September 2021) and the first-year postnatal surveys under approval No. KICCEIRB-2022-01 (28 January 2022). This study analyzed de-identified data from the panel, and additional ethical approval was therefore not required.

## 3. Results

### 3.1. Sociodemographic Characteristics and Antenatal Depressive Symptoms

As shown in Table 1, the prevalence of antenatal depressive symptoms at Baseline 1 varied by sociodemographic characteristics. Screen-positive status was less frequent in twin than in singleton pregnancies (2 of 69, 2.9%, versus 348 of 3209, 10.8%; odds ratio (OR) 0.25, 95% CI 0.06–1.01), although this estimate rests on two events and no inference should be drawn from it. The prevalence was higher among women living in rural townships than among those in metropolitan areas (OR 1.57, 95% CI 1.14–2.15), whereas the difference between mid-sized/small cities and metropolitan areas was not significant (OR 1.05, 95% CI 0.82–1.34). Women in low-income households were more likely to screen positive than other women (OR 2.40, 95% CI 1.31–4.39).

Of the 3278 women in the analytic sample, 2447 were assessed at the first annual follow-up and 831 were not. The two groups did not differ in K-EPDS score at Baseline 1 (5.28 versus 5.33; *p* = 0.74), maternal age (32.9 versus 32.9 years; *p* = 0.94), parity, pregnancy intention or pre-pregnancy diagnosis of depression; women who were retained were somewhat more likely to have university education (64.8% versus 59.3%; *p* = 0.015). A comparable distribution of baseline scores does not exclude attrition related to depressive symptoms arising later in follow-up, and we therefore also report an inverse-probability-weighted sensitivity analysis.

### 3.2. Antenatal and Postpartum Depressive Symptoms by Time Point

Table 2 presents the distribution of K-EPDS scores at the four assessments. The proportion screening positive rose from 10.68% at Baseline 1 (16–31 weeks of gestation) to 18.54% at Baseline 2 (32–37 weeks), 26.51% at one month postpartum and 28.08% at the first annual follow-up. Because these proportions are based on different denominators, they are described rather than tested here; within-person change is tested with the generalized estimating equation model reported below.

Scores at the four assessments were substantially correlated within women (Table S2): Pearson correlations were 0.559 between Baseline 1 and Baseline 2 (*n* = 2945), 0.394 between Baseline 1 and one month postpartum (*n* = 2716), 0.426 between Baseline 1 and the first annual follow-up (*n* = 2447), 0.548 between Baseline 2 and one month postpartum (*n* = 2653), 0.557 between Baseline 2 and the first annual follow-up (*n* = 2335) and 0.600 between one month postpartum and the first annual follow-up (*n* = 2273); all *p* < 0.0001.

**Figure 2:**
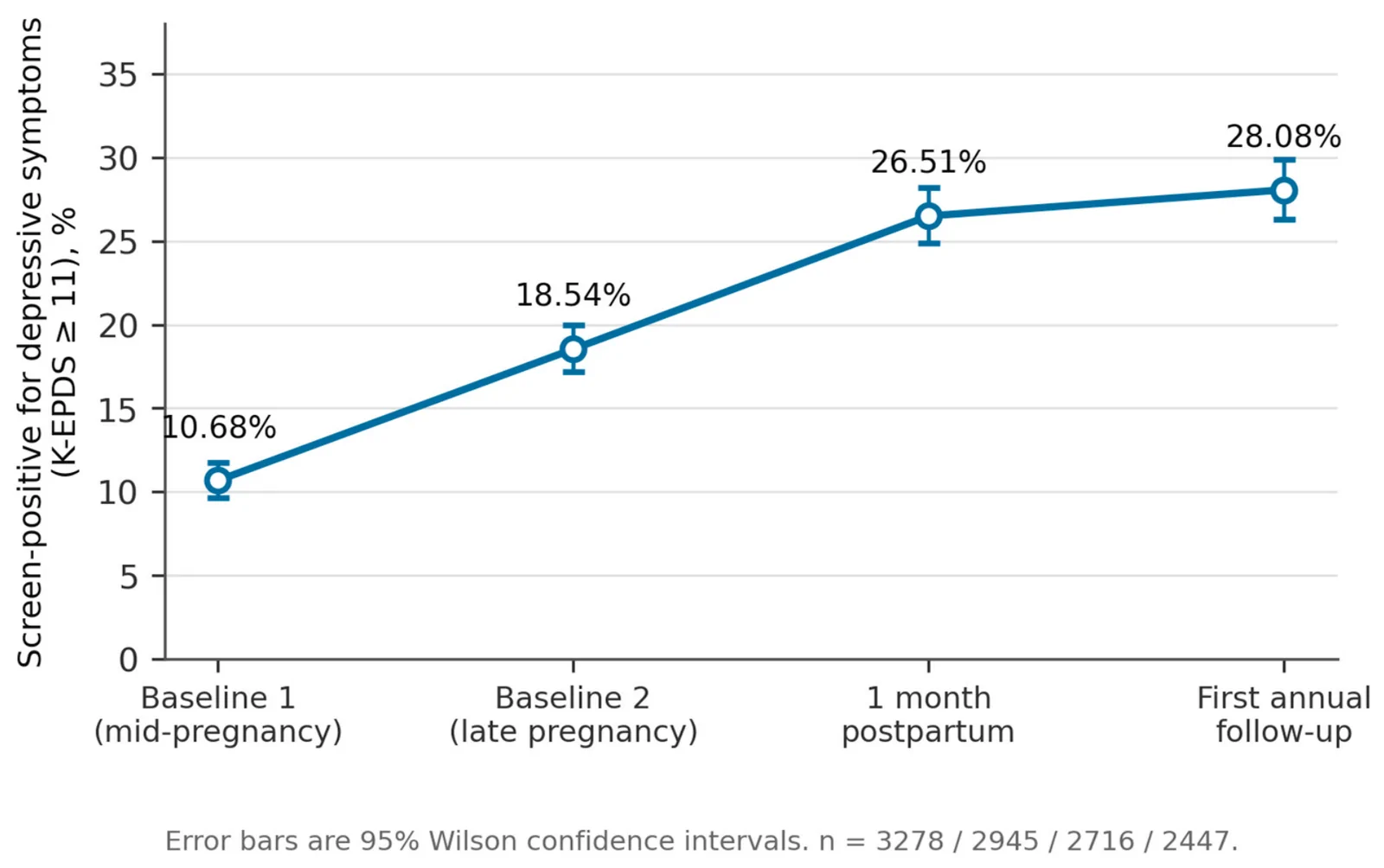
Prevalence of screen-positive depressive symptoms (K-EPDS ≥ 11) at each of the four assessments. Error bars are 95% Wilson confidence intervals.

Change over time was tested with a generalized estimating equation model comprising 9502 observations from 2447 women (estimated working correlation 0.35). With assessment number entered linearly, the odds of screening positive increased by a factor of 1.46 per successive assessment (95% CI 1.41–1.53; *p* < 0.0001). With assessment number entered categorically and Baseline 1 as the reference, the odds ratios were 1.81 (95% CI 1.60–2.06) at Baseline 2, 3.08 (95% CI 2.69–3.53) at one month postpartum and 3.35 (95% CI 2.92–3.83) at the first annual follow-up, a monotonic pattern consistent with the linear specification. Neither the main effect of absence of reported knowledge on maternal–neonatal interaction (OR 1.01, 95% CI 0.64–1.60; *p* = 0.96) nor its interaction with assessment number (OR 1.09, 95% CI 0.95–1.25; *p* = 0.21) was statistically significant. The prevalence of screen-positive depressive symptoms across the four assessments is shown in Figure 2.

### 3.3. Factors Associated with Postpartum Depressive Symptoms

Table 3 presents factors associated with the onset of postpartum depressive symptoms. Among women who screened negative at Baseline 1 (K-EPDS < 11; *n* = 2191), we compared those who screened positive at the first annual follow-up with those who did not.

In unadjusted analysis, women aged under 30 years were more likely to screen positive than older women (28.9% versus 22.5%; OR 1.40, 95% CI 1.10–1.78), and women with twin pregnancies were more likely to do so than those with singleton pregnancies (41.9% versus 23.2%; OR 2.39, 95% CI 1.43–4.00). Absence of reported knowledge on maternal–neonatal interaction was also associated with the outcome (30.6% versus 23.1%; OR 1.47, 95% CI 1.06–2.04; *p* = 0.021); the other three knowledge domains were not.

Because these estimates are unadjusted, we fitted a multivariable model in the same subgroup, entering all four knowledge domains simultaneously together with antenatal K-EPDS score at Baseline 2, maternal age, parity, household income, residential area, plurality, pregnancy intention, a pre-pregnancy diagnosis of depression, social support and maternal education (*n* = 2037 with complete covariate data). After adjustment, none of the four domains was associated with the outcome: adjusted odds ratios were 1.32 (95% CI 0.58–3.04) for newborn care, 0.97 (95% CI 0.67–1.40) for maternal health management, 1.10 (95% CI 0.67–1.80; *p* = 0.71) for maternal–neonatal interaction and 0.77 (95% CI 0.51–1.18) for prevention of sudden infant death. After Benjamini–Hochberg correction across the four domains, all adjusted *p* values were 0.87.

In the same model, social support (OR 0.64 per point, 95% CI 0.54–0.76; *p* < 0.0001), antenatal K-EPDS score (OR 1.22 per point, 95% CI 1.18–1.25; *p* < 0.0001), twin pregnancy (OR 2.61, 95% CI 1.45–4.68; *p* = 0.001) and maternal age under 30 years (OR 1.40, 95% CI 1.04–1.89; *p* = 0.028) were associated with screening positive. Results were materially unchanged when the threshold was set at ≥ 10 (adjusted OR for maternal–neonatal interaction 1.38, 95% CI 0.85–2.23) and when inverse-probability-of-participation weights were applied (weighted adjusted OR 1.09, 95% CI 0.66–1.82).

Refitting the same model in the same 2037 women with the continuous K-EPDS score as the outcome gave the same conclusion. None of the four domains was associated with the score; the adjusted difference for maternal–neonatal interaction was +0.01 points (95% CI −0.87 to +0.89; *p* = 0.98). Social support (−1.08 points per point, 95% CI −1.38 to −0.77), antenatal K-EPDS score (+0.58 points per point, 95% CI +0.54 to +0.63) and a pre-pregnancy diagnosis of depression (+1.71 points, 95% CI +0.19 to +3.24) were associated with higher scores.

**Table 3:** Comparison of precipitating factors between the two groups (with and without postpartum depressive symptoms at the first annual follow-up) in those who have no depressive mood at Baseline 1.

Variable		Total	With Postpartum Depressive Symptoms		Without Postpartum Depressive Symptoms		CrudeOR	95% CI	Adjusted OR	95% CI
			n	Rate (%)	n	Rate (%)				
Plurality	Singleton	2129	494	23.20	1635	76.80	0.418	0.250–0.700	0.383	0.214–0.688
	Twin	62	26	41.94	36	58.06				
Residential area	Metropolitan	879	195	22.18	684	77.82				
	Mid-sized/small city	1025	253	24.68	772	75.32	1.150	0.929–1.423	1.111	0.870–1.420
	Rural	287	72	25.09	215	74.91	1.175	0.861–1.603	0.970	0.672–1.398
Maternal age	<30 years	415	120	28.92	295	71.08	1.470	1.143–1.891	1.402	1.038–1.893
	30–35 years	1232	267	21.67	965	78.33				
	≥36 years	544	133	24.45	411	75.55	1.170	0.922–1.484	0.951	0.721–1.253
Household income	Middle/high	2152	506	23.51	1646	76.49				
	low	39	14	35.90	25	64.10	1.832	0.941–3.534	1.629	0.742–3.577
Neonatal care	No experience	49	17	34.69	32	65.31	1.732	0.954–3.146	1.321	0.575–3.035
	Without partner participation	1206	277	22.97	929	77.03	0.937	0.766–1.145		
Maternal health care	No experience	316	87	27.53	229	72.47	1.265	0.967–1.656	0.971	0.674–1.398
	Without partner participation	1114	248	22.26	866	77.74	0.892	0.717–1.108		
Maternal–neonatal interaction	No experience	186	57	30.65	129	69.35	1.473	1.060–2.044	1.097	0.670–1.795
	Without partner participation	1046	243	23.23	803	76.77	1.017	0.826–1.334		
SIDS prevention	No experience	260	61	23.46	199	76.54	0.983	0.724–1.334	0.774	0.507–1.181

Rate (%) indicates the proportion of women with a K-EPDS score ≥11. Adjusted odds ratios were obtained from a single multivariable logistic regression model (*n* = 2037) that additionally included antenatal K-EPDS score at Baseline 2 (continuous), parity, pregnancy intention, pre-pregnancy diagnosis of depression, social support, maternal education, and the sudden-infant-death-prevention education domain; estimates for social support, antenatal K-EPDS score and maternal education are reported in the text. Partner participation was not included because it is defined only among women who received the corresponding education. Abbreviations: OR, odds ratio; CI, confidence interval.

### 3.4. Factors Associated with the Remission of Postpartum Depressive Symptoms

Among women who screened positive at Baseline 1 (K-EPDS ≥ 11) and were reassessed at the first annual follow-up (*n* = 256), we examined factors associated with remission. The subgroup is small, so these analyses are exploratory and have limited power. As shown in Table 4, remission was not associated with any of the knowledge domains.

Remission was more frequent with increasing maternal age: it occurred in 12 of 47 women aged under 30 years (25.5%), 39 of 133 aged 30–35 years (29.3%) and 38 of 76 aged 36 years or older (50.0%). Taking women aged 30–35 years as the reference and remission as the outcome, the odds ratio was 0.83 (95% CI 0.39–1.76; *p* = 0.62) for women aged under 30 years and 2.41 (95% CI 1.34–4.32; *p* = 0.003) for women aged 36 years or older. The odds ratios shown in Table 4 are expressed on the opposite scale: they describe the odds of remaining screen-positive at the first annual follow-up relative to the reference category shown, so the value of 2.92 for women aged under 30 years is relative to women aged 36 years or older. An earlier version of this manuscript described the direction of this association incorrectly, and the text has been corrected.

Only two women with twin pregnancies were included in this subgroup, and the corresponding estimate is therefore uninformative (OR 0.53, 95% CI 0.03–8.58; Firth penalised estimate 0.53, 95% CI 0.03–8.60); no inference regarding twin pregnancy can be drawn.

**Table 4:** Comparison of remission factors between the two groups (with and without postpartum depressive symptoms at the first annual follow-up) in those who have depressive mood at Baseline 1.

Variable		Total	With Postpartum Depressive Symptoms		Without Postpartum Depressive Symptoms		OR	95% CI
			n	Rate (%)	n	Rate (%)		
Plurality	Singleton	254	166	65.35	88	34.65		
	Twin	2	1	50	1	50	0.530	0.033–8.578
Residential area	Metropolitan	96	61	63.54	35	36.46	0.697	0.331–1.470
	Mid-sized/small city	111	71	63.96	40	36.04	0.710	0.342–1.475
	Rural	49	35	71.43	14	28.57		
Maternal age	<30 years	47	35	74.47	12	25.53	2.916	1.317–6.457
	30–35 years	133	94	70.68	39	29.32	2.410	1.344–4.324
	≥36 years	76	38	50	38	50		
Household income	Middle/high	246	160	65.04	86	34.96	0.797	0.201–3.162
	Low	10	7	70	3	30		
Neonatal care	Reported any source	249	163	65.46	86	34.54	1.422	0.311–6.497
	With partner participation	90	61	67.78	29	32.22	1.175	0.678–2.034
Maternal health care	Reported any source	218	143	65.6	75	34.4	1.112	0.544–2.276
	With partner participation	64	47	73.44	17	26.56	1.670	0.878–3.178
Maternal–neonatal interaction	Reported any source	237	157	66.24	80	33.76	1.766	0.690–4.521
	With partner participation	96	62	64.58	34	35.42	0.883	0.511–1.525
SIDS prevention	Reported any source	229	151	65.94	78	34.06	1.331	0.589–3.006
	With partner participation	87	58	66.67	29	33.33	1.054	0.599–1.853

Rate (%) indicates the proportion of women with a K-EPDS score ≥11. Abbreviations: OR, odds ratio; CI, confidence interval. Outcome modeled: K-EPDS ≥ 11 at the first annual follow-up (that is, failure to remit). Reference categories: singleton pregnancy, rural residence, maternal age 36 years or older, low household income; for the knowledge domains, having reported no source for that topic; and for partner participation, non-attendance by the spouse, assessed only among women who reported a formal source. Odds ratios are Wald estimates with 95% confidence intervals. The subgroup contains only two twin pregnancies and no inference regarding twin pregnancy can be drawn.

### 3.5. Depressive Mood During Pregnancy and Subsequent Childbirth Intention

As shown in Table 5, women who screened positive were less likely to report an intention for a further birth at every assessment. The association was not statistically significant at Baseline 1 (OR 1.32 for reporting no intention, 95% CI 0.99–1.75; *p* = 0.055) and was significant at Baseline 2 (OR 1.62, 95% CI 1.27–2.07), at one month postpartum (OR 1.50, 95% CI 1.22–1.84) and at the first annual follow-up (OR 1.94, 95% CI 1.59–2.37; all *p* < 0.001).

**Figure 3:**
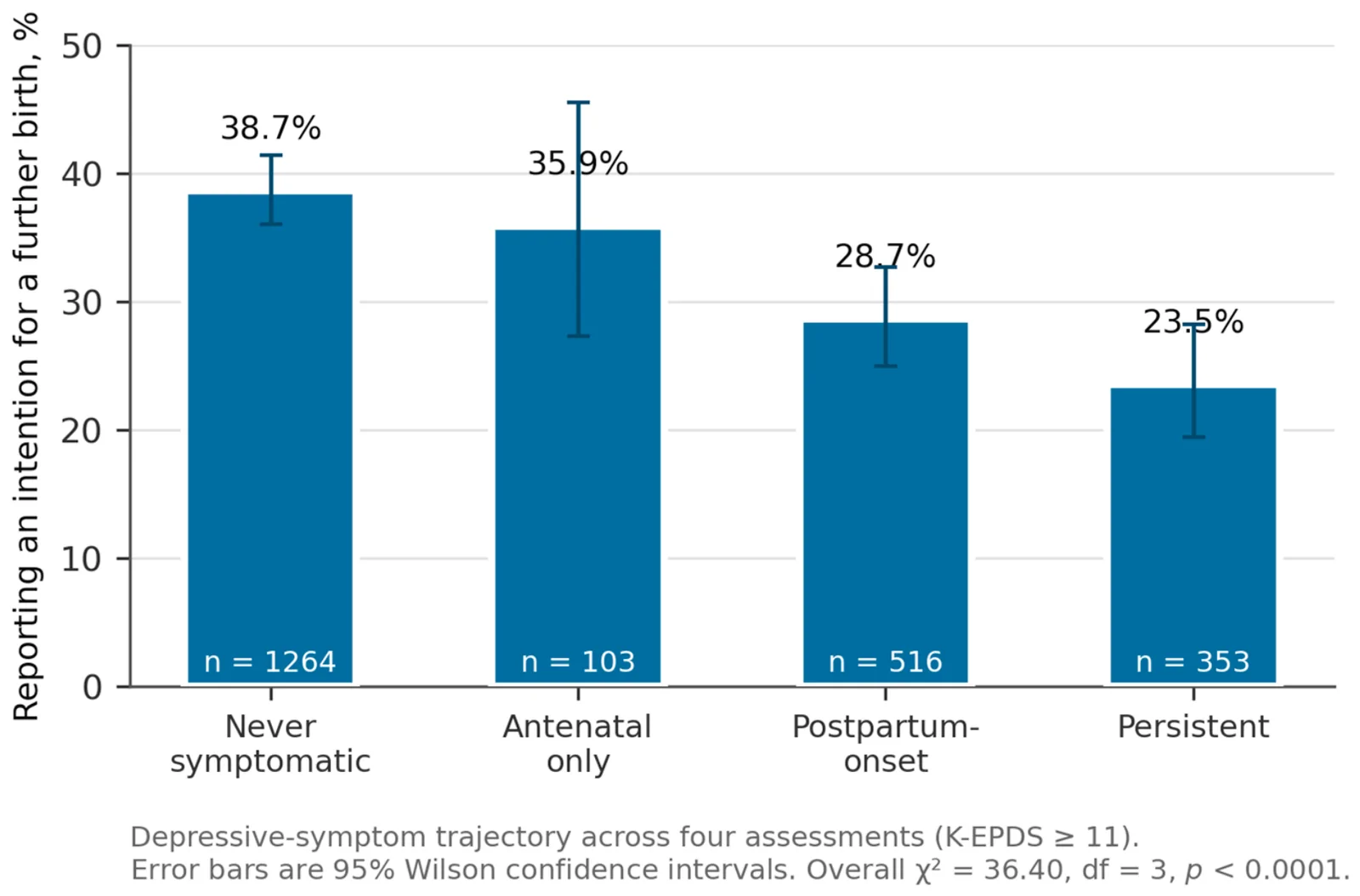
Proportion reporting an intention for a further birth by depressive-symptom trajectory (*n* = 2236). Error bars are 95% Wilson confidence intervals; overall χ^2^ = 36.40, df = 3, *p* < 0.0001.

Table 6 shows childbirth intention according to the trajectory of depressive symptoms. The sixteen possible patterns were consolidated into four categories: a woman was classified as having antenatal symptoms if she screened positive at Baseline 1 or Baseline 2 and as having postpartum symptoms if she screened positive at one month postpartum or at the first annual follow-up; the categories are never symptomatic, antenatal symptoms only, postpartum-onset and persistent. “Persistent” therefore denotes screen-positive status in both periods and does not require a positive screen at all four assessments. Among the 2236 women assessed at all four time points, the proportion reporting an intention for a further birth was 38.69% of 1264 never-symptomatic women, 35.92% of 103 with antenatal symptoms only, 28.68% of 516 with postpartum-onset symptoms and 23.51% of 353 with persistent symptoms (χ^2^ = 36.40, df = 3, *p* < 0.0001). In a model adjusted for maternal age, household income, parity, social support, maternal education and pregnancy intention, women with postpartum-onset symptoms and women with persistent symptoms were less likely than never-symptomatic women to report an intention for a further birth, whereas women with antenatal symptoms only did not differ. These comparisons are exploratory and are not corrected for multiplicity. These differences are shown in Figure 3.

**Table 5:** Association between antenatal and postpartum depressive symptoms and subsequent childbirth intention.

Depressive Symptoms		Total	Childbirth Intention		No Childbirth Intention		*p*-Value	OR	95% CI
			n	Rate (%)	n	Rate (%)			
Baseline 1 (Antenatal)	Present	256	74	28.91	182	71.09	0.055	1.319	0.993–1.753
	Absent	2191	765	34.92	1426	65.08			
Baseline 2 (Antenatal)	Present	405	103	25.43	302	74.57	<0.0001	1.620	1.272–2.064
	Absent	1930	687	35.6	1243	64.4			
1 month Postpartum	Present	597	164	27.47	433	72.53	<0.0001	1.499	1.221–1.841
	Absent	1676	607	36.22	1069	63.78			
First annual follow-up	Present	687	166	24.16	521	75.84	<0.0001	1.943	1.592–2.372
	Absent	1760	673	38.24	1087	61.76			

Abbreviations: OR, odds ratio; CI, confidence interval. *p* values are from Pearson’s chi-square test without continuity correction. Odds ratios are for reporting no intention for a further birth, with screen-negative women as the reference.

**Table 6:** Intention for a further birth according to depressive-symptom trajectory.

Antenatal Depressive Symptoms (Baseline 1)	Antenatal Depressive Symptoms (Baseline 2)	Postpartum 1 Month Depressive Symptoms	First Annual Follow-Up Depressive Symptoms	Total (2236)	Childbirth Intention *		No Childbirth Intention ^†^	
					n	Rate (%)	n	Rate (%)
Present	Present	Present	Present	100	22	22	78	78
			Absent	11	2	18.18	9	81.82
		Absent	Present	19	6	31.58	13	68.42
			Absent	24	7	29.17	17	70.83
	Absent	Present	Present	21	7	33.33	14	66.67
			Absent	13	4	30.77	9	69.23
		Absent	Present	9	2	22.22	7	77.78
			Absent	32	14	43.75	18	56.25
Absent	Present	Present	Present	85	16	18.82	69	81.18
			Absent	45	14	31.11	31	68.89
		Absent	Present	50	10	20	40	80
			Absent	47	16	34.04	31	65.96
	Absent	Present	Present	126	26	20.63	100	79.37
			Absent	183	68	37.16	115	62.84
		Absent	Present	207	54	26.09	153	73.91
			Absent	1264	489	38.69	775	61.31

Trajectory categories were derived from the four assessments as follows: a woman was classified as having antenatal symptoms if she screened positive at Baseline 1 or Baseline 2, and as having postpartum symptoms if she screened positive at one month postpartum or at the first annual follow-up. The four categories are never symptomatic (neither period), antenatal symptoms only (antenatal but not postpartum), postpartum-onset (postpartum but not antenatal) and persistent (both periods). Applying this rule to the sixteen patterns above gives 1264 never-symptomatic women, 103 with antenatal symptoms only, 516 with postpartum-onset symptoms and 353 with persistent symptoms (*n* = 2236). “Persistent” therefore denotes screen-positive status in both periods and does not require a positive screen at all four assessments. * Childbirth intention: intends to have another child. ^†^ No childbirth intention: does not intend to have another child.

## 4. Discussion

Given South Korea’s persistently low birth rate and aging population, addressing maternal mental health has become central not only to improving women’s health but also to promoting national demographic sustainability [1,13,14]. In this context, postpartum depressive symptoms are a critical concern with implications that extend beyond individual health outcomes to impact reproductive choices and demographic trends. While previous studies have established that PPD can impair maternal role, child development, and parent-infant bonding [7,14], limited research has examined how self-reported acquisition of perinatal knowledge relates to depressive symptoms within a nationally representative population.

Drawing on the nationally representative longitudinal Korean ECEC Panel, this study addresses previously underexplored gaps and offers an important contribution to the advancement of the literature. The results indicate that women who reported never having learned about maternal–neonatal interaction were more likely to screen positive for depressive symptoms in unadjusted analysis, but that this association did not persist after adjustment. A substantial body of literature has explored the factors contributing to PPD, including lack of social support, economic hardship, past psychiatric history, and obstetric complications [4,5,14]. Recently, increasing attention has been directed toward prenatal education and psychological preparedness for the maternal role as potential protective factors that may buffer against the onset of PPD. Prior research indicates that prenatal education programs can reduce maternal anxiety, improve birth preparedness, and enhance maternal self-efficacy, all of which may contribute to lowering the risk of postpartum depressive symptoms [8,15]. In particular, structured prenatal education has been shown to significantly reduce EPDS scores at six weeks postpartum and to increase maternal confidence [15,16]. However, it remains unclear whether these protective effects are consistently observed at the population level. This gap is especially salient given the rapid demographic shifts occurring in South Korea, including rising maternal age at first birth and declining fertility rates, underscoring the need for analyses using nationally representative samples.

Alizadeh-Dibazari et al. reported that structured prenatal education reduces fear of childbirth and lowers the risk of postpartum psychological disorders [8]. Our unadjusted estimates are in the same direction, but the association did not persist after adjustment, and our exposure records the source of knowledge rather than participation in a structured program, so the two are not directly comparable. A null result in a cohort of this size does not establish the absence of an association, and the confidence intervals remain compatible with modest associations in either direction. What the data do not support is the interpretation offered in our original submission, namely that acquiring perinatal knowledge lowers the risk of postpartum depressive symptoms at the population level. The exposure was recorded at a median child age of six months, concurrently with the final outcome assessment, so the temporal ordering required for such an interpretation is not established, and women who reported never having learned about a topic already had higher antenatal depressive symptom scores and lower social support. The clinical weight of this persistence is illustrated by Duan et al., who reported in 23,218 Japanese mother–child pairs that maternal distress during pregnancy and depressive symptoms postpartum were each associated with an increased risk of autistic-related traits in toddlers, with the strongest associations in girls exposed during both periods [10].

The most consistent finding of the adjusted analyses was the inverse association between social support and postpartum depressive symptoms, which was present on both the binary and the continuous scale and was of a magnitude comparable with that of the antenatal symptom score. This is consistent with the existing literature [17] and, unlike the knowledge variables, it is measured on a validated multi-item scale. This also offers a plausible explanation for the attenuation of the association with reported knowledge after adjustment: women with fewer support resources may have had both fewer opportunities to acquire perinatal knowledge and a higher risk of depressive symptoms. These findings underscore the importance of considering social support when examining the relationship between perinatal knowledge acquisition and depressive symptoms.

Consistent with Shim et al., who emphasized psychosocial stressors and maternal self-rated health as dominant predictors of PPD in the Korean context [14], our findings do not identify self-reported acquisition of perinatal knowledge as an independent correlate of postpartum depressive symptoms once antenatal symptoms and social support are accounted for. While Shim et al. focused on vulnerability stemming from health and stress perception, our adjusted analyses point in the same direction as theirs: the strongest correlates in our data were antenatal symptom severity and social support rather than the knowledge variables.

This study is distinct from previous research in that it utilizes data from the Korean ECEC Panel, a nationally representative longitudinal dataset. The strength of this dataset lies in its ability to trace maternal mental health during perinatal period, from mid-pregnancy to the first annual follow-up, while accounting for a broad range of sociodemographic and psychosocial variables. The Korean ECEC Panel tracks maternal and infant outcomes across pre- and postnatal stages using a national sample, enhancing the generalizability of findings [18]. This approach enables a more dynamic understanding of how self-reported acquisition of perinatal knowledge and maternal experiences intersect to shape postpartum mental health and fertility planning.

A notable finding of this study is that mothers of multiple births exhibit a substantially higher risk of developing postpartum depressive symptoms at the first annual follow-up compared with mothers of singletons. This is consistent with prior evidence indicating that the physical and emotional demands of caring for twins or higher-order multiples—particularly during the early postpartum period—can intensify stress and fatigue, thereby increasing vulnerability to depressive symptoms [7]. These results highlight the need for targeted support mechanisms, such as home-based postnatal care and respite services, to better address the unique challenges faced by mothers of multiple births.

Maternal age showed two patterns that point in opposite directions. Among women who screened negative at Baseline 1, those aged under 30 years were the most likely to screen positive at the first annual follow-up (28.9% versus 21.7% at 30–35 years and 24.5% at 36 years or older; adjusted OR 1.40, 95% CI 1.04–1.89 relative to women aged 30–35 years). Among women who screened positive during pregnancy, by contrast, remission was more frequent with increasing age: half of those aged 36 years or older had remitted by the first annual follow-up, compared with roughly a quarter of those aged under 30 years. An earlier version of this manuscript reported the age association in the opposite direction, which was an error in the interpretation of the odds ratios rather than in their calculation. Because the remission subgroup comprises 256 women and neither analysis was prespecified, we present both as exploratory and do not speculate on mechanism. These observations suggest that maternal age may be relevant to perinatal risk assessment, but the two patterns differ by age group and by outcome, and neither was prespecified.

In addition to identifying risk factors, this study examined the association between depressive-symptom trajectories and maternal intention regarding a further birth. Women with postpartum-onset or persistent depressive symptoms were less likely to report an intention for a further birth than never-symptomatic women, whereas women with antenatal symptoms only did not differ significantly. These findings suggest an association between the timing and persistence of depressive symptoms and subsequent childbirth intention; however, childbirth intention was assessed at a single time point and should not be interpreted as subsequent fertility behavior.

Furthermore, this study underscores the compounding effect of depression duration. Mothers classified as having persistent depressive symptoms across the antenatal and postpartum periods had the lowest proportion reporting an intention for a further birth. In contrast, those with less persistent symptom patterns more often reported such an intention. Previous studies have similarly identified parenting burden, economic concerns, and perceived lack of support as factors associated with decisions regarding additional childbirth [19,20]. These findings should be interpreted as associations and do not establish that improvement in depressive symptoms would alter subsequent reproductive behavior.

This study has several methodological strengths. First, we used a nationally representative longitudinal dataset (Korean ECEC Panel) with four time points across the perinatal period, allowing for a comprehensive examination of depressive symptom trajectories to the first annual follow-up. Second, Baseline 1 and Baseline 2 assessments were appropriately classified as antenatal depressive symptoms rather than PPD, given that they were measured prior to delivery. Furthermore, this study incorporated the antenatal K-EPDS score at Baseline 2 as a covariate in the adjusted model, acknowledging that prior depressive symptoms are among the strongest established predictors of PPD. This approach reduces confounding and enhances the validity of estimates for other predictors such as self-reported acquisition of perinatal knowledge and twin pregnancy.

### Study Limitations

Several limitations should be acknowledged. First, the exposure was recorded at the first annual follow-up, at a median child age of six months, and the instrument recorded where knowledge was obtained rather than participation in a defined educational programme; the content, format, duration and quality of any education received cannot be characterised. The temporal ordering of exposure and outcome therefore cannot be established, and reverse causation cannot be excluded, since women with antenatal depressive symptoms may be less likely to seek or receive such information. Women who did and did not report each source may also differ systematically in baseline mental health, pregnancy planning, socioeconomic circumstances and healthcare access, and adjustment cannot be assumed to remove these differences. Second, although social support, antenatal symptom severity and a pre-pregnancy diagnosis of depression were included as covariates, other psychosocial factors established in the literature as predictors of postpartum distress—notably parenting stress and the quality of the partner relationship—are not available in the panel and remain unmeasured confounders. Third, the analyses are unweighted and do not incorporate the panel’s sampling or attrition weights, which limits generalisation of the absolute prevalence estimates to the national population. Fourth, 831 of 3278 women were not assessed at the first annual follow-up; although the two groups were comparable in baseline symptoms and an inverse-probability-weighted analysis gave similar estimates, attrition related to symptoms arising later in follow-up cannot be excluded. Fifth, the K-EPDS is a screening instrument, and the outcome is screen-positive status rather than clinically confirmed depression. The Korean translation was validated in postpartum women, and the threshold of 11 or higher is taken from an individual participant data meta-analysis of the original scale rather than from a Korean validation study; the operating characteristics of the Korean version during pregnancy and at this specific threshold have therefore not been separately established. Sixth, the subgroup analysis of remission included only two women with twin pregnancies, and no inference regarding twin pregnancy can be drawn from it. Seventh, intention regarding a further birth is a self-reported attitude recorded at a single time point; although childbirth intention has been reported to predict subsequent births, our data do not include subsequent pregnancies or deliveries, and no inference about fertility or demographic outcomes can be drawn. Finally, the primary exposure and outcome and the four trajectory categories were specified during revision of this manuscript rather than before the original analysis, and all analyses other than the primary association are exploratory; the false discovery rate correction was applied only across the four knowledge domains and not to the other comparisons reported. Future studies with prospectively measured educational participation and with parenting stress and partner relationship quality would allow these questions to be addressed more directly.

## 5. Conclusions

In this nationally representative Korean cohort, the proportion of mothers screening positive for depressive symptoms rose from roughly one in ten at mid-pregnancy to more than one in four at the first annual follow-up. Three findings carry implications for perinatal care. First, postpartum depressive symptoms were not a transient phenomenon: antenatal symptom severity was the strongest correlate of later symptoms, and women whose symptoms persisted across the antenatal and postpartum periods were the least likely to intend a further birth. Screening should therefore begin in pregnancy and extend well beyond the early postpartum weeks, when routine contact with maternity services has usually ended. Second, perinatal knowledge in Korea is acquired largely outside formal health services—from postpartum care centres, the internet and family—and a substantial minority of mothers reported no source at all for some topics. Self-reported acquisition of such knowledge was not independently associated with depressive symptoms once antenatal symptoms and social support were taken into account, but the channels through which mothers currently obtain information represent an under-used opportunity to deliver structured, evidence-based content and to identify women who need support. Third, social support showed the most consistent inverse association with depressive symptoms, suggesting that interventions which strengthen support networks may matter at least as much as the provision of educational content. Because the design is observational, these findings identify priorities for prospective evaluation rather than establishing that perinatal education prevents depressive symptoms. Nonetheless, in a country facing very low fertility, the association between persistent postpartum depressive symptoms and reduced intention for a further birth makes sustained perinatal mental health care a public health as well as a clinical priority.

## Figures and Tables

**Table 1:** Sociodemographic characteristics associated with antenatal depressive symptoms (Baseline 1).

Variable		Total	With Antenatal Depressive Symptoms		Without Antenatal Depressive Symptoms		OR	95% CI
			n	Rate (%)	n	Rate (%)		
Plurality	Singleton	3209	350	10.68	2928	89.32		
	Twin	69	2	2.9	67	97.1	0.245	0.060–1.006
Residential area	Metropolitan	1285	126	9.81	1159	90.19		
	Mid-sized/small city	1526	156	10.22	1370	89.78	1.047	0.818–1.341
	Rural	467	68	14.56	399	85.44	1.568	1.143–2.150
Maternal age	<30 years	643	69	10.73	574	89.27	1.051	0.784–1.408
	30–35 years	1792	184	10.27	1608	89.73		
	≥36 years	843	97	11.51	746	88.49	1.136	0.875–1.475
Household income	Middle/high	3214	336	10.45	2878	89.55		
	low	64	14	21.88	50	78.13	2.399	1.312–4.385

Abbreviations: OR, odds ratio; CI, confidence interval. Statistical significance was set at *p* < 0.05. Reference categories: singleton pregnancy, metropolitan residence, maternal age 30–35 years, middle/high household income. *p* values are from Pearson’s chi-square test without continuity correction.

**Table 2:** Prevalence of antenatal and postpartum depressive symptoms across four time points (Baseline 1, Baseline 2, one month postpartum, and the first annual follow-up).

	n	Mean (SD)	Median (IQR)	K-EPDS ≥ 11, n (%)
Baseline 1 (16–31 weeks)	3278	5.29 (4.04)	5 (3–7)	350 (10.68)
Baseline 2 (32–37 weeks)	2945	6.45 (4.77)	5 (3–9)	546 (18.54)
One month postpartum	2716	7.57 (5.36)	7 (3–11)	720 (26.51)
First annual follow-up	2447	7.76 (5.69)	7 (3–11)	687 (28.08)

Rate (%) indicates the proportion of women with a K-EPDS score ≥ 11. Values are for the whole sample at each assessment. Abbreviations: SD, standard deviation; IQR, interquartile range. Within-person change across assessments is tested by the generalized estimating equation model reported in Section 3.2; the proportions are based on different denominators and are not themselves a test of change over time.

## Data Availability

The data analyzed in this study were obtained from the Korea Institute of Child Care and Education (KICCE) under a data-use agreement and are available to researchers on application through the KICCE panel data portal (https://panel.kicce.re.kr). The analysis code is available from the corresponding author on request.

## Supplementary Materials

The following supporting information can be downloaded at https://www.mdpi.com/article/10.3390/epidemiologia7050145/s1: Table S1: reported source of perinatal knowledge by content domain; Table S2: Pearson correlations between K-EPDS scores at the four assessments.
