# Supplementary material for: The Association Between Self-Reported Acquisition of Perinatal Knowledge and Perinatal Depressive Symptoms: Evidence from the Korean Early Childhood Education & Care Panel Study
Source: Epidemiologia (Basel). 2026 Oct 15;7(5):145. doi: 10.3390/epidemiologia7050145 (PMC13702919; doi:10.3390/epidemiologia7050145)
Supplement: Supplementary file 1 [file epidemiologia-07-00145-s001.zip › epidemiologia-4508677-supplementary.pdf]

Supplementary Materials

The Association Between Self-Reported Acquisition of Perinatal Knowledge and Perinatal Depressive Symptoms: Evidence from the Korean Early Childhood Education & Care Panel Study

Source	Newborn care	Maternal health management	Interaction with the newborn	Prevention of sudden infant death
**Hospital**	323 (13.2)	697 (28.5)	174 (7.1)	414 (16.9)
**Midwifery clinic or postpartum care centre**	1,557 (63.6)	744 (30.4)	744 (30.4)	595 (24.3)
**Public health centre or healthy family support centre**	29 (1.2)	29 (1.2)	48 (2.0)	66 (2.7)
**Childcare support centre**	3 (0.1)	2 (0.1)	2 (0.1)	7 (0.3)
**Publicly funded postpartum helper service**	120 (4.9)	91 (3.7)	167 (6.8)	45 (1.8)
**Private postpartum helper**	24 (1.0)	17 (0.7)	26 (1.1)	10 (0.4)
**Own parents**	77 (3.1)	78 (3.2)	102 (4.2)	40 (1.6)
**Parents-in-law**	3 (0.1)	1 (0.0)	2 (0.1)	2 (0.1)
**Siblings**	8 (0.3)	11 (0.4)	17 (0.7)	10 (0.4)
**Internet or books**	247 (10.1)	423 (17.3)	960 (39.2)	971 (39.7)

**Never learned about this topic**	56 (2.3)	354 (14.5)	205 (8.4)	287 (11.7)

Table S1. Reported source of perinatal knowledge, by content domain (n = 2,447)

Values are n (%). Responses were recorded at the first annual follow-up survey for each of the four content domains. The exposure analysed in the manuscript is the final row ("never learned about this topic") versus all other responses. Percentages are column percentages and may not sum to 100 because of rounding.

	**Baseline 2**	**One month postpartum**	**First annual follow-up**
**Baseline 1**	0.559 (n = 2,945)	0.394 (n = 2,716)	0.426 (n = 2,447)
**Baseline 2**	—	0.548 (n = 2,653)	0.557 (n = 2,335)
**One month postpartum**	—	—	0.600 (n = 2,273)

Table S2. Pearson correlations between K-EPDS scores at the four assessments

All correlations P < 0.0001. n is the number of women with a K-EPDS score at both assessments. Within-person change across assessments is tested with the generalized estimating equation model reported in Section 3.2.
